# Down-regulation of HSPA9 reduces tyrosine hydroxylase-positive neurons in mouse substantia nigra and induces Parkinson’s disease-like motor impairments

**DOI:** 10.1080/19768354.2025.2569875

**Published:** 2025-10-14

**Authors:** Hyejin Hyung, Soyoung Jang, Si-Yong Kim, Ji-Eun Bae, Ji Yeong Park, Su-Geun Lim, Jiwon Ko, Soyeon Jang, Joon Bum Kim, Hee Young Chae, Song Park, Junkoo Yi, Dong Kyu Choi, Myoung Ok Kim, Hyun-Shik Lee, Dong-Hyung Cho, Zae Young Ryoo

**Affiliations:** a School of Life Sciences, BK21 FOUR KNU Creative BioResearch Group, Kyungpook National University, Daegu, Republic of Korea; b Division of Animal Bioscience and Integrated Biotechnology, Gyeongsang National University, Jinju, Republic of Korea; c College of Agriculture and Life Sciences, Gyeongsang National University, Jinju, Republic of Korea; d Organelle Institute, Kyungpook National University, Daegu, Republic of Korea; e Core Protein Resources Center, DGIST, Daegu, Republic of Korea; f Division of Animal Science, Gyeongsang National University, Jinju, Republic of Korea; g Institute of Agriculture and Life Science (IALS), Gyeongsang National University, Jinju, Republic of Korea; h School of Animal Life Convergence Science, Hankyong National University, Anseong, Korea; i School of Animal Science Biotechnology, Kyungpook National University, Sangju, Republic of Korea

**Keywords:** HSPA9, Parkinson’s disease, movement disorder, mitochondrial dysfunction

## Abstract

Parkinson’s disease (PD) is a progressive neurological disorder characterized by the degeneration of midbrain dopaminergic neurons and disabling motor impairments. Heat shock protein family A member 9 (HSPA9) play a crucial role in neuronal homeostasis by regulating the import of various mitochondrial proteins. HSPA9 is down-regulated in neurodegenerative diseases such as Alzheimer’s disease and PD, and its loss leads to excessive mitochondrial fragmentation with oxidative stress, which subsequently causes damage to dopaminergic neurons. Moreover, HSPA9 interacts with multiple PD-associated proteins, including Pink1, DJ-1, and α-synuclein, however precise roles of HSPA9 in PD pathophysiology remain unclear. To further explore the contributions of HSPA9 in PD pathogenesis, we developed an HSPA9 knockout mouse. Haploinsufficiency of Hspa9 (*Hspa9*^+/−^) was associated with the loss of tyrosine hydroxylase-positive neurons in the striatum and substantia nigra. Furthermore, *Hspa9* haploinsufficiency induced excessive mitochondrial fission, enhanced apoptotic signaling, and resulted in diminished motor performance during the rotarod test. Administration of the mitochondrial neurotoxin 1-methyl-4-phenyl-1,2,3,6-tetrahydropyridine (MPTP) in *Hspa9*^+/−^ mice further exacerbated the loss of dopaminergic neurons, aggravated motor impairments, and enhanced activation of apoptosis effector caspase-3. These results suggest that down-regulation of HSPA9 may contribute to the development and progression of PD, potentially offering a new therapeutic strategy for PD treatment.

## Introduction

Parkinson’s disease (PD) is a neurodegenerative disorder that afflicts over five million individuals globally. The core pathology of PD is loss of dopaminergic neurons in the substantia nigra (SN), resulting in dopamine deficiency in the striatum (STR) (Ferreira and Romero-Ramos 2018; Goloborshcheva et al. 2022; Hurben and Tretyakova 2022) and various motor symptoms, including muscle rigidity, resting tremor, postural instability, slowness of voluntary movement, and bradykinesia (Mallet et al. 2019). In addition, PD causes a range of other physical and psychiatric symptoms, including swallowing difficulties (dysphagia), sleep disorders, depression, anxiety, and dementia in later stages.

Heat shock protein family A member 9 (HSPA9 also known as mortalin or GRP75) is mainly localized in mitochondria but also found in the endoplasmic reticulum, plasma membrane, and cytosolic vesicles (Lee et al. 2017). As a chaperon protein, HSPA9 is activated by cellular stress, including oxidative stress, metabolic stress, and glucose deprivation, but not by heat shock stress (Texier et al. 2023). Several studies have reported that HSPA9 insufficiency impairs mitochondrial function, morphology, and subsequently leads to oxidative stress, metabolic failure, and neuronal injury (Yang et al. 2011; Park et al. 2014; Bae et al. 2023). In the central nervous system, HSPA9 protects against neuronal damage caused by misfolded or aggregated proteins, and helps to import proteins into the mitochondria (Zhu et al. 2013; Lv et al. 2017). Additionally, HSPA9 prevents the accumulation of amyloid-β and α-synuclein in mitochondria and promotes proper protein folding or degradation (Priyanka 2022). HSPA9 expression is reduced in the PD brain due to covalent modification by dopamine under conditions of dopamine toxicity and mitochondrial damage (Gabriele et al. 2010; Chiasserini et al. 2011).

Mitochondrial capacity is maintained at the cellular and subcellular levels by the dynamic balance between fission and fusion. Dynamin-related protein 1 (Drp1) is a crucial regulator of mitochondrial fission (Hu et al. 2017). Phosphorylation of Drp1 at Ser 616 results in mitochondrial translocation and fission, whereas phosphorylation at Ser 637 inhibits Drp1 activity (Cereghetti et al. 2010; Zerihun et al. 2023). Excessive mitochondrial fragmentation heightens susceptibility to pro-apoptotic stimuli (Kim and Kang 2018), and dopaminergic neurons are particularly susceptible to this form of programed cell death (Li et al. 2022). In fact, mitochondrial dysfunction in neurons is a common feature of *in vitro* and *in vivo* models of neurodegenerative diseases such as PD, Alzheimer's disease (AD), and Huntington’s disease (HD) models (Pozo Devoto and Falzone 2017; Norat et al. 2020). The vast majority of PD case (>90%) are sporadic (idiopathic), although a distinct subset of cases has an autosomal monogenic origin, primarily mutations in the genes encoding α-synuclein, Parkin, PTEN-induced kinase 1 (Pink1), deglycase J-1 (DJ-1), and leucine-rich repeat kinase 2 (LRRK2). These mutant proteins have been found to disrupt mitochondrial quality control, impair autophagy, induce oxidative stress, and trigger neuroinflammation, collectively accelerating the progression of PD (Kasten et al. 2018). Thus, mitochondrial defects are the primary drivers of pathology in familial as well as sporadic PD (Santos et al. 2015).

Exposure to 1-methyl-4-phenyl-1,2,3,6-tetrahydropyridine (MPTP) can induce a PD-like syndrome in both experimental animals and humans (Mat Taib and Mustapha 2020). MPTP is converted to the metabolically active form 1-methyl-4-phenylpyridinium (MPP^+^) by monoamine oxidase B (AlShimemeri et al. 2022), and is then selectively internalized by dopaminergic neurons via dopamine transporters, where it inhibits respiratory chain complex I, leading to metabolic failure and excessive generation of reactive oxygen species (ROS) (Borsche et al. 2021). Although many of the clinical and pathological characteristics simulated by MPTP differ from those of PD, both conditions involve inhibition of respiratory chain complexes, mitochondrial dysfunction, and ROS-induced oxidative stress. Therefore, MPTP has been used to induce a mitochondrial toxin model that mimics PD.

Our group recently reported that the depletion of Hspa9 in a neuronal cell line exacerbated Aβ-mediated mitochondrial dysfunction and induced excessive peroxisomal ROS, which led to peroxisomal autophagy (Park et al. 2014; Jo et al. 2020). Reduced expression of HSPA9 has also been detected in patients with PD as well as in 6-hydroxydopamine (6-OHDA) rat and *Drosophila* models (Gabriele et al. 2010). However, there is no direct evidence to show associations among HSPA9 deficiency, dopaminergic neuronal death, and motor performance impairments. Therefore, we generated a haploinsufficient *Hspa9* (*Hspa9*^+/−^) mouse and examined the effects on dopaminergic neuron survival, mitochondrial fission markers, and motor performance.

## Materials and methods

### Animals

Mice were house under controlled temperature (20°C–22°C), humidity (50%–60%), and light/dark cycle (12 h/12 h) with free access to food and water. *Hspa9* knockout mice were established using the CRISPR/Cas9 system on the C57BL/6J genetic background. The sequences of small guide RNAs (sgRNAs) for *Hspa9* knockout mice were 5′-CATGTGTTTCTCAGAATAATTGG-3′ and 5′-CCTTGATCACTTTTTCATTATTT-3′, and founder mice were identified by polymerase chain reaction (PCR). Offspring genotype was confirmed by PCR targeting wild-type (WT) and heterozygous (*Hspa9^+/−^*) alleles using genomic DNA extracted from 4-week-old mouse ears. PCR amplification was performed with Hspa9 primers (Forward: 5’-CACACATGGGGTTTTGTGTGC-3’, Reverse: 5’-CTGGGAAGGCAAAAATGGGCT-3’), which produced 432-bp fragments. The body weights of WT and *Hspa9^+/−^* mice were measured once weekly from 4 to 10 weeks of age.

### Mouse embryonic fibroblast (MEF) cell culture and treatment

MEF cells were established from WT and *Hspa9^+/−^* embryos as previously described (Ferreira and Hein 2023). MEF cells were cultured in Dulbecco’s Modified Eagle’s Media (DMEM, Gibco) supplemented with 15% Fetal Bovine Serum (Gibco) and 1% Penicillin–Streptomycin (Gibco) at 37°C in an 5% CO_2_. All experiments were performed using early-passage MEF cells (passages 2–4) to avoid replicative senescence.

### Measurement of mitochondrial length

MEF cells were fixed with 4% PFA and then treated with a MitoTracker probe (100 nM, M7512, Thermo Fisher Scientific, MA, USA) for 30 min. All images were visualized using a fluorescence microscope (IX71; Olympus, Japan). The mitochondrial length was quantified via the Cell Sense Standards software (Olympus Europa Holding GmbH).

### Assessment of mitochondrial membrane potential

MEF cells were seeded in 12-well plates and grown to 70–80% confluence. Liquiritigenin was treated with 20 µM for 24 h. Mitochondrial membrane potential was assessed using the fluorescent dye tertramethylrhodamine ethyl ester (TMRE; T669, Thermo Fisher Scientific), which accumulates in active mitochondria in a membrane potential-dependent manner. After treatment, cells were incubated with 200 nM TMRE in pre-warmed complete medium for 30 min at 37 °C in the dark, washed gently with PBS, and immediately analyzed. Fluorescence intensity was detected using a fluorescence (IX71; Olympus, Japan). Images were quantified as mean fluorescence intensity using ImageJ software.

### MPTP-induced PD mouse model

Mice were randomly separated into four groups, control WT, MPTP-treated WT, control *Hspa9^+/−^*, and MPTP-treated *Hspa9^+/−^*. An acute intoxication procedure was used to examine the contribution of *Hspa9* deficiency to PD pathology in 9- to 10-week-old male *Hspa9^+/−^* mice and age-matched WT littermates. To construct this model, mice were injected intraperitoneally (i.p.) with 20 mg/kg MPTP hydrochloride (S4732, Selleck Chemicals LLC., TX, USA) in 0.9% saline four times at 2-h intervals within a single day (for a total dose of 80 mg/kg) (Jackson-Lewis and Przedborski 2007). Behavioral assessments were conducted and body weight changes assessed 25 h after the first injection. Animals were then sacrificed 25 h after the last dose of MPTP for gene expression, protein expression, and immunohistochemical analyses (described below). Control groups were administered an equal amount of normal saline. MPTP handling and safety measures were in accordance with published guidelines (Kasahara et al. 2017).

### Mouse behavioral tests

All tests were performed in a dark room after 30 min of acclimatization, and the animals were trained on the task the day before testing.

#### Rotarod test

To assess forelimb and hindlimb motor coordination and balance, the rotarod test was performed on 6- to 7-week-old and 9- to 10-week-old male WT and *Hspa9^+/−^* mice. Mice were acclimated to the rotarod apparatus (SK-RO02MR, B.S Technolab INC., Seoul, Korea) by placing them on the rotating rod and conducting preliminary trials before starting data collection. The rotarod was programed to rotate at a linearly increasing speed from 4 to 40 rpm in 300 s. The latency to fall off the rod as speed increased was measured in three separate trials conducted at least 10 min apart and averaged for each mouse.

#### Tail suspension test

The tail suspension test was performed to evaluate dyskinesia (diminished voluntary movement) and the stage of disease progression. Briefly, mice were suspended in the air by the tail and hindlimb clasping (contacting the abdomen) was observed for 60 s (Guyenet et al. 2010; Liu et al. 2014).

#### Footprint test

To evaluate stepping abnormalities, the forelimbs and hindlimbs of mice were painted with red or green nontoxic paints, respectively. The animals were then allowed to walk along a 50 cm long, 5.5 cm wide, and 5 cm high runway into an enclosed box. Each mouse was tested twice. To characterize the walking pattern of each mouse, we measured the stride length (SL) and forepaw/hindpaw overlap. The SL for both hindlimbs was measured by calculating the average distance of forward movement over at least 5 steps per animal. The forepaw/hindpaw overlap was analyzed by measuring the distance between prints on the same side. All parameters were measured (in cm) from the center of each pawprint (Carter et al. 1999).

#### Pole test

The pole test was used to assess basal ganglia-related movement dysfunction (bradykinesia or slowness of movement). Animals were positioned with their head upward near the top of a 60 cm rough-surfaced wooden pole with a diameter of 1 cm. The time required for the mouse to reorient its body completely downward from the top of the pole was defined as the ‘T-turn,’ while the time taken to descend from the point of turning to the cage floor was defined as the ‘T-descend’ (Balkaya et al. 2013). The test was repeated three times per animal, and the average was calculated. Both parameters (T-turn and T-descend) are validated metrics for bradykinesia in mice (Stojakovic et al. 2019).

### Real-time PCR (qRT–PCR)

Total mRNA was extracted from mouse brain tissue using TRI-Solution (TS100-001, BSK-BIO Technology, Daegu, Korea) according to the manufacturer’s instructions and reverse transcribed into cDNA using the PrimeScript 1st strand cDNA Synthesis Kit (6110A, TaKaRa Bio Inc., Shiga, Japan). A 500–1000 ng sample of total RNA was used for each assay. The primer sequences used for target gene detection were as follows: *Hspa9*: forward (5’-GTTGGTATGCCAGCAAAACGGC-3’) and reverse (5’-CAAGCATCACCATTGGAGGCAC-3’); and β-actin: forward (5’-TCTGGCACCACACCTTCTACA-3’) and reverse (5’-TTTTCACGGTTGGCCTTAGG-3’). qRT-PCR was performed using a QuantaStudio Real-Time PCR System (Applied Biosystems) with SYBR Premix Ex Taq (RR420A, TaKaRa Bio Inc.). Gene expression was estimated using the $2^{-\Delta \Delta C_{T}}$ method and β-actin expression was measured as an internal control.

### Western blot analysis

Western blotting was performed on brain tissue samples from 8- to 9-week-old male mice. Lysates were prepared using PRO-PREP Protein Extraction Solution (17081, iNtRON Biotechnology, Inc., Seongnam, Korea) containing protease inhibitors (P3100-001, GenDEPOT, Texas, USA). The lysates were centrifuged at 13,000 rpm for 15 min at 4°C, and the total protein concentration in the supernatant was measured. Equal amounts of protein (20 µg per gel lane) were separated on 10% SDS-PAGE gels at constant voltage and then transferred to PVDF membranes (Millipore Sigma, Billerica, MA, USA). Membranes were blocked in TBS containing Tween 20 (TBS-T) plus 5% skim milk (MB Cell, Los Angeles, CA, USA) for 30 min at room temperature, then incubated overnight at 4°C in TBS-T containing anti-Hspa9 (818801, BioLegend, San Diego, USA), anti-tyrosine hydroxylase (TH) (58844, Cell Signaling Technology, Danvers, MA, USA), and anti-β-actin (sc-47778, Santa Cruz, Dallas, TX, USA). Immunoreactivity was visualized using West-Q Pico Dura ECL Solution (W3653, GenDEPOT). β-actin expression was measured as an internal control.

### Histological analysis

Animals were anesthetized with avertin (200 mg/kg) and transcardially perfused with PBS (pH 7.4). Brains were removed, fixed in 4% paraformaldehyde/PBS, embedded in paraffin, and sectioned using a microtome (HM355, Thermo Scientific). For immunofluorescence staining, sections were incubated with primary antibodies against HSPA9 (818801, BioLegend), cleaved caspase-3 (9661, Cell Signaling Technology, Danvers, MA, USA), and p-Drp1 (Ser 616) (3455, Cell Signaling Technology) at 4°C overnight, followed by incubation with Alexa Fluor-488- and Alexa Fluor-555-conjugated secondary antibodies at room temperature for 2 h. Images were captured using a fluorescence microscope (Leica DMI3000B, Leica Microsystems Ltd.). For immunohistochemistry, coronal sections were incubated sequentially in an antibody against TH (58844, Cell Signaling Technology) at 4°C overnight and a horseradish peroxidase (HRP)-conjugated secondary antibody for 1 h at room temperature. Immunolabeling was visualized using diaminobenzidine (DAB). Sections were counterstained with hematoxylin and detected using a Carl Zeiss Axio Scan.Z1 microscope (Oberkochen, Germany).

### Statistical analysis

All results are expressed as mean values. Two groups were compared by independent samples *t*-tests followed by nonparametric Kolmogorov–Smirnov tests. Changes in body weight (%change) and behavioral test outcomes were compared by two-way ANOVA with main factors genotype and treatment, followed by Tukey’s multiple comparison tests for pairwise comparisons. GraphPad Prism 10 software (San Diego, CA, USA) was used for all statistical analysis, and *p* < 0.05 was considered statistically significant for all tests. In figures, levels of significance are indicated by ns *p* > 0.05, *p < 0.05, **p < 0.01, and ***p < 0.001.

## Results

### Down-regulation of Hspa9 influences the degeneration of dopaminergic neurons through mitochondrial dysfunction in an Hspa9^+/ –^ mouse model

**Figure 1.**
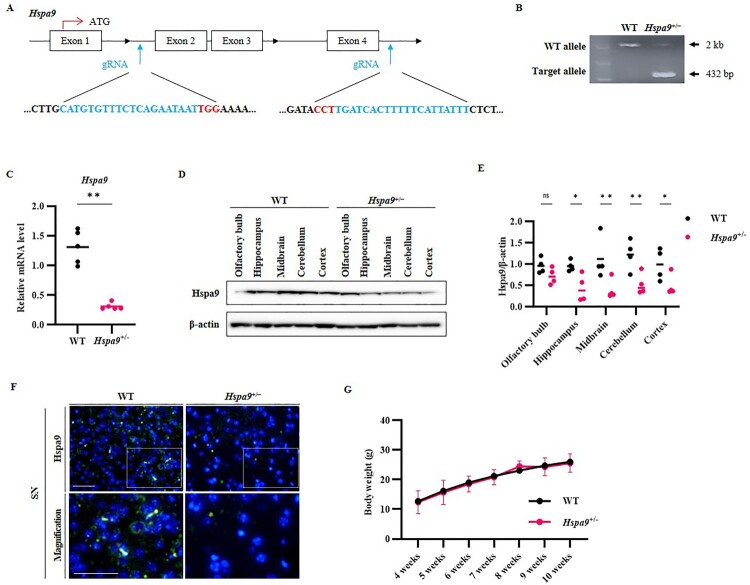
CRISPR/Cas9-mediated generation of Hspa9 deficient mice. (A) *Hspa9* knockout mice were generated by targeting exons 2–4 in the murine *Hspa9* gene region using the CRISPR/Cas9 system. The sgRNA sequences are marked in blue and the PAM sequences in red. A span of 329 bp was deleted from exons 2–4. (B) Representative PCR genotyping results for WT and *Hspa9*^+/−^ mice. (C) Comparison of whole-brain *Hspa9* mRNA expression levels between *Hspa9*^+/−^ mice and WT littermates (normalized to β-actin levels; n = 5 mice per group). (D and E) The level of Hspa9 expression was analyzed by western blotting (n = 4). (F) Immunofluorescence staining of Hspa9 in brain sagittal sections (green) confirming reduced expression in *Hspa9*^+/−^ mice. Nuclei were counterstained with DAPI (blue) (n = 3). Scale bar, 20 μm. (G) Knockdown of *Hspa9* had no effect on mouse body weight from 4 to 10 weeks of age (n = 5 mice per group). All results are expressed as the values of three independent experiments. **p *<* 0.01.

To investigate the potential contributions of *Hspa9* insufficiency to PD pathogenesis, we generated a transgenic mouse using the CRISPR/Cas9 system to target exons 2–4 in the *Hspa9* gene region (Figure 1A). A total of 1665 bp were removed, including the intron region, with 329 bp being deleted from the exons 2–4 region, and genotypes were confirmed by PCR analysis (Figure 1B). The intercrossing of *Hspa9* heterozygous mice resulted in no homozygous offspring, suggesting the homozygous depletion of Hspa9 is embryonic lethal on the C56BL/6J background. Thus, all experiments were conducted using haplodeficient heterozygous (*Hspa9*^+/−^) mice. Down-regulation of *Hspa9* expression in the whole brain tissue of *Hspa9*^+/−^ mice was confirmed at the mRNA level by qRT–PCR (Figure 1C) and at the protein level by western blotting and immunofluorescence staining (Figure 1D,F). We confirmed that Hspa9 is localized to mitochondria in WT MEF cells via immunofluorescence imaging (Supplementary Figure 1A and B). As shown in Figure 1G, the reduction of Hspa9 caused no significant change in body weight from 4 to 10 weeks of age compared to WT littermates (Figure 1G).

**Figure 2.**
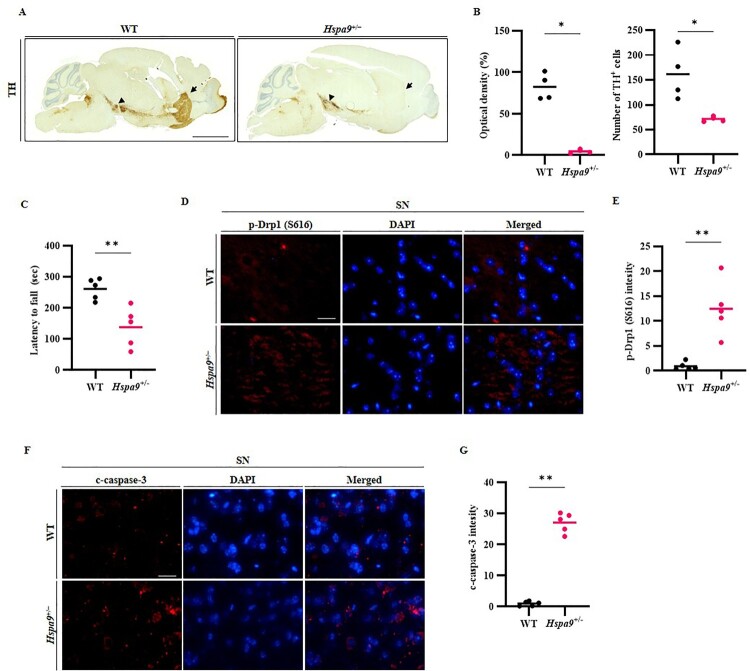
Depletion of Hspa9 induces dopaminergic neuron degeneration by accelerating mitochondrial fission and activating caspase-3. (A) Immunohistochemical staining of tyrosine hydroxylase (TH) was used to determine dopaminergic neurons in the STR (arrow) and SN (arrowhead) from WT and *Hspa9*^+/−^ mice. Scale bar, 2000 μm. (B and C) *Hspa9*^+/−^ mice exhibited significant motor deficits in the rotarod test at 6–7 weeks of age as indicated by shorter mean latency to fall (n = 5 mice per group). (D and E) Immunofluorescence staining of p-Drp1 (Ser 616) (red) indicating accelerated mitochondrial fission in the SN of *Hspa9*^+/−^ mice compared to WT littermates. Scale bar, 20 μm. (F and G) Enhanced activation of the apoptosis effector c-caspase-3 (red) in the SN of *Hspa9*^+/−^ mice compared to WT. Nuclei were counterstained with DAPI (blue). Scale bar, 20 μm. Results are expressed as the mean values of three independent experiments. **p *<* 0.01.

The expression of TH is reduced in the nigrostriatal region of PD patients and various animal models (Daubner et al. 2011; Campos et al. 2013; Li et al. 2021), reflecting the loss of dopaminergic neurons. Further, TH can induce neuronal oxidative stress through direct interactions with chaperone proteins (Daubner et al. 2011). Thus, we examined immunohistochemical staining revealed that TH-positive region was substantially lower in the STR and SN of *Hspa9*^+/−^ mice compared to WT mice (Figure 2A,B), indicating that *Hspa9* haploinsufficiency induced dopaminergic neuron degeneration. To investigate whether the reduction in dopaminergic input from the SN to the STR disrupted motor control (as observed in PD patients and PD animal models), WT and *Hspa9*^+/−^ mice were compared for rotarod performance. The latency to fall was significantly shorter among *Hspa9*^+/−^ mice than WT mice (Figure 2C), indicating that the observed dopaminergic neuron loss was sufficient to induce motor dysfunction.

Given the established connection between mitochondrial damage and PD progression, we then examined if *Hspa9* haploinsufficiency altered the expression of mitochondrial function markers. The expression of Drp1 phosphorylated at Ser 616 (p-Drp1), which promotes mitochondrial fission, was slightly enhanced, as was the expression of the apoptotic effector cleaved caspase-3 (c-caspase-3). Thus, reduced *Hspa9* expression may have enhanced mitochondrial fission within the SN via p-Drp1 (Ser 616), resulting in an accelerated rate of apoptosis (Figure 2D,E). To further explore the cellular mechanisms underlying *Hspa9* haploinsufficiency, we conducted *in vitro* experiments using mouse embryonic fibroblast (MEF) cells. Knockdown of Hspa9 markedly increased mitochondrial fission (Supplementary Figure 2A). Moreover, treatment with liquiritigenin significantly restored membrane potential (ΔΨm), as assessed by TMRE fluorescence in *Hspa9*^+/−^ MEF cells (Supplementary Figure 2B), consistent with its previously reported ability to promote mitochondrial fusion and suppress fragmentation (Jo et al. 2016).

To verify potential neuronal injury resulting from *Hspa9* haploinsufficiency, mouse brain sections were immunostained for c-caspase-3. Immunoexpression of c-caspase-3 was significantly greater in brain sections from *Hspa9*^+/−^ mice compared to WT mice (Figure 2F,G), further supporting the notion that *Hspa9* deficiency can induce TH-positive cell death, driven by the activation of caspase-3 triggered by mitochondrial fission.

### Hspa9 knockdown exacerbates MPTP-induced motor impairment

**Figure 3.**
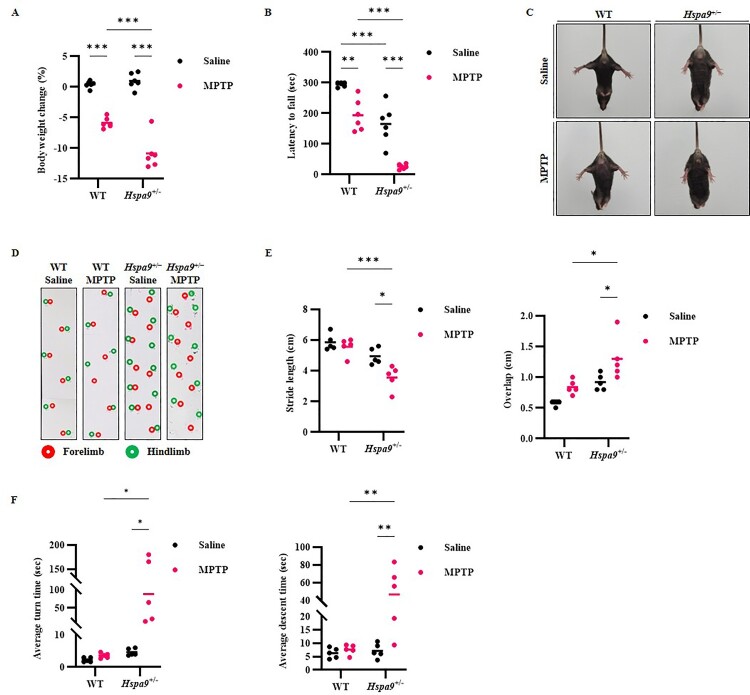
Knockdown of Hspa9 aggravates MPTP-induced motor dysfunction. (A) Body weight changes was confirmed 25 h after injection of WT and *Hspa9*^+/−^ mice with MPTP (red) or saline (black). MPTP injection induced marked body weight loss in *Hspa9*^+/−^ mice but not WT mice. (B) MPTP exacerbated motor coordination deficits to a greater extent in *Hspa9*^+/−^ mice than WT mice as indicated by the larger relative decrease in latency to fall during rotarod testing. (C) MPTP exacerbated signs of dyskinesia (absence of hind limb splaying) in *Hspa9*^+/−^ mice but not WT mice during tail suspension. (D) MPTP exacerbated walking abnormalities in *Hspa9*^+/−^ mice but not WT mice. (E) Quantification of the hindlimb stride length and overlap of the footprint patterns. (F) MPTP increased the time-to-turn and descent time of *Hspa9*^+/−^ but not WT mice during the pole test. Results are expressed as the mean values of three independent experiments (n = 5). The Y-axis is discontinuous to emphasize the separation between clustered low values and dispersed higher responses. *p* > 0.05, *p < 0.05, **p < 0.01, ***p < 0.001.

To investigate if *Hspa9* haploinsufficiency can increase the susceptibility of dopaminergic neurons to MPTP toxicity, we compared motor performances among WT and *Hspa9*^+/−^ mice administered MPTP or equal-volume saline (controls). Post-injection of MPTP induced a significant body weight change in *Hspa9*^+/−^ mice compared to MPTP-treated WT mice, while no difference was observed between saline-treated WT and *Hspa9*^+/−^ mice (Figure 3A). Acute MPTP treatment markedly reduced the latency to fall in both *Hspa9*^+/−^ mice and WT compared to corresponding saline-treated controls, whereas the proportionate reduction was markedly greater in MPTP-injected *Hspa9*^+/−^ mice. In contrast, no significant difference in latency to fall was observed between MPTP-injected WT and saline-injected *Hspa9*^+/−^ mice (Figure 3B). In the tail suspension test, WT mice displayed a normal hindlimb extension reflex, while *Hspa9*^+/−^ mice tended to retract the hindlimbs slightly toward the abdomen (clasping). Following MPTP injection, however, *Hspa9*^+/−^ mice exhibited full hindlimb clasping with abdominal contact while WT mice showed only minor hindlimb retraction without abdominal contact (Figure 3C). Taken together, these results indicate that *Hspa9* down-regulation can exacerbate MPTP-induced dyskinesia.

Patients with PD also tend to have difficulty walking, taking small steps with their feet close together in order to maintain balance. To examine if *Hspa9* haploinsufficiency can exacerbate walking abnormalities induced by MPTP, we compared SL and forepaw/hindpaw overlap calculated from walking records. SL, defined as the distance between each step taken on the same side of the body (Kasahara et al. 2013), was reduced in MPTP-injected *Hspa9*^+/−^ mice compared to corresponding saline-injected *Hspa9*^+/−^ mice, while little change was noted in WT mice. In addition, overlap as measured by the distance between the center of the forepaw and hindpaw prints on the same side, was enhanced by MPTP injection in *Hspa9*^+/−^ mice but not WT mice (Figure 3D,E). Lastly, in the pole test used to assess motor deficits related to basal ganglia dysfunction in mouse models of PD, both T-turn and T-descend times were longer in MPTP-injected *Hspa9*^+/−^ mice than MPTP-injected WT mice (Figure 3F). Collectively, these findings indicate that *Hspa9* haploinsufficiency can increase susceptibility to MPTP-induced motor dysfunction, possibly by exacerbating the degeneration of dopaminergic neurons.

### Depletion of Hspa9 exacerbates MPTP-induced loss of dopaminergic neurons

**Figure 4.**
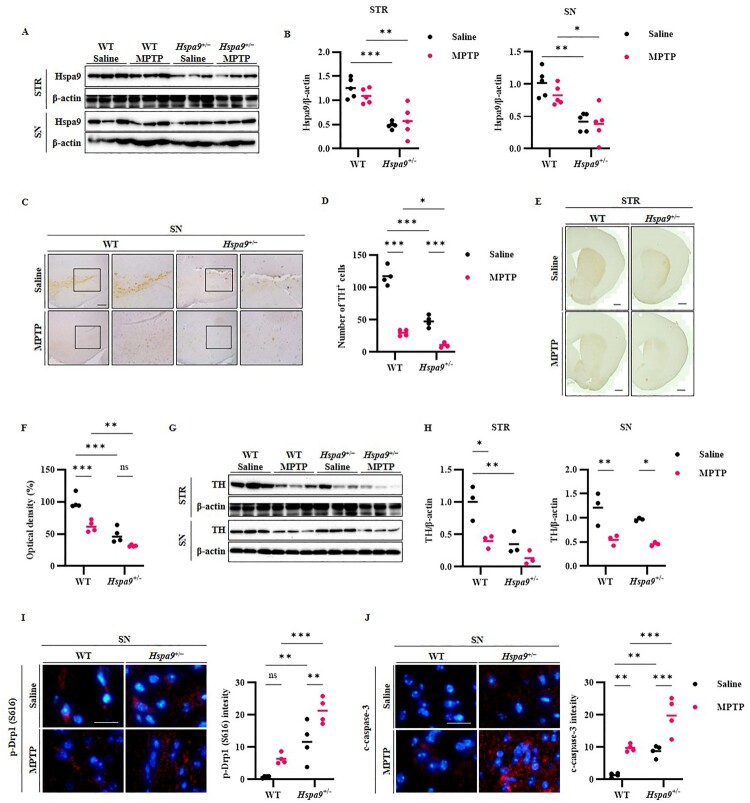
Down-regulation of Hspa9 exacerbates MPTP-mediated degeneration of dopaminergic neurons. (A) Western blots estimating Hspa9 expression changes in the STR and SN of *Hspa9*^+/−^ mice and WT mice following MPTP injection. (B) Quantified analysis of Western blotting images is represented graphically. (C) Immunohistochemical staining of TH illustrating reduced dopaminergic cell in the SN following MPTP injection. The reduction was far greater in the SN of *Hspa9*^+/−^ mice than WT mice. Scale bar, 100 μm. (D) The number of TH positive cells in SN. (E) Immunohistochemical staining of TH in the STR showing qualitatively similar results. Scale bar, 1000 μm. (F) The intensity of TH in STR. (G) Western blot analysis of TH in the STR and SN. (H) Quantified analysis of Western blotting images is represented graphically. (I) Immunofluorescence staining of p-Drp1 (Ser 616) (red) with DAPI counterstaining (blue) in the SN of *Hspa9*^+/−^ mice and WT mice following MPTP injection. Scale bar, 20 μm. The intensity of p-Drp1 (Ser 616) was analyzed. (J) Immunostaining for c-caspase-3 in the SN showing enhanced expression in *Hspa9*^+/−^ mice compared to WT mice following MPTP injection. Scale bar, 20 μm. The intensity of c-caspase-3 was measured. Results are expressed as the mean values of three independent experiments. ns *p* > 0.05, *p < 0.05, **p < 0.01, ***p < 0.001.

Following the behavioral tests, we conducted histological analyses to assess the changes in TH-labeled neurons induced by reduced Hspa9 expression, MPTP injection, and both combined. Treatment with MPTP or MPP^+^ has been reported to increase Hspa9 expression in mouse brain and SH-SY5Y cells (Cai et al. 2022). Thus, we first compared Hspa9 expression levels in the STR and SN of *Hspa9*^+/−^ and WT mice injected with MPTP or saline, but found no significant differences between MPTP-treated and corresponding control mice (Figure 4A,B). Nonetheless, MPTP injection reduced the number of TH-labeled dopaminergic cells in the STR and SN of *Hspa9*^+/−^ mice to a greater extent than in WT mice (Figure 4B–H). Moreover, we found that the greater loss of TH-labeled neurons was associated with larger increases in p-Drp1 (Ser 616) and c-caspase-3 expression within the SN (Figure 4I,J). These findings suggest that *Hspa9* haploinsufficiency leads to excessive mitochondrial fission and concomitant activation of caspase-3, resulting in greater apoptotic death of dopaminergic neurons. Moreover, down-regulation of Hspa9 results in heightened sensitivity to MPTP-induced neurotoxicity.

## Discussion

To develop effective treatments for neurodegenerative diseases, it is essential to identify the most promising molecular targets, a task greatly aided by *in vitro* and *in vivo* models that recapitulate important aspects of disease pathogenesis (Luo et al. 2020). In this study, we provide evidence that insufficient *Hspa9* expression in brain can promote the apoptotic death of dopaminergic neurons in the STR and SN by enhancing mitochondrial fission. Moreover, we demonstrate that this accelerated dopaminergic neuronal death can manifest as motor deficits resembling those of PD. Therefore, enhancement of HSPA9 expression may be an effective therapeutic strategy for slowing PD progression.

Previous studies have highlighted that the deficiency of Hspa9, coupled with mitochondrial oxidative stress, contributes to the progression of PD pathology (Texier et al. 2023). In the current study, we generated the haploinsufficiency of *Hspa9* (*Hspa9*^+/−^) and examined the effect of its depletion on PD-related pathology. Notably, young adult *Hspa9*^+/−^ mice (7 weeks old) show diminished TH-labeled neurons and induced PD-like motor impairments even in the absence of the neurotoxin MPTP. Loss of TH-positive neurons was associated with enhanced expression of the mitochondrial fission marker p-Drp1 (Ser 616), which can promote apoptotic cell death via activation of caspase-3 (Figure 2). The study revealed exacerbated mitochondrial dysfunction in *Hspa9*^+/−^ mice, a recognized factor in PD-related neurodegeneration. This was evidenced by increased mitochondrial fission marker p-Drp1 (Ser 616) and elevated c-caspase-3 levels, indicating apoptotic cell death (Imbeault et al. 2014).

Moreover, *Hspa9* deficiency exacerbated the neurotoxicity and motor deficits observed following injection of MPTP, a compound that can induce mitochondrial dysfunction and a PD-like syndrome in both humans and animals (AlShimemeri et al. 2022). Further, this exacerbation was not accompanied by a significant change in *Hspa9* expression but was associated with significant increases in p-Drp1 (Ser 616) and c-caspase-3 expression in the SN, providing further support for the associations among *Hspa9* insufficiency, excessive mitochondrial fission, and dopaminergic neuronal death in this mouse model.

Recent studies have reported a significant decrease in striatal dopaminergic terminals and SN dopaminergic neurons two days after acute MPTP challenge (Rabaneda-Lombarte et al. 2021). In contrast, *Hspa9*^+/−^ mice exhibited significant decreases in both regions after just one day, resulting in more rapid-onset motor deficits. Considering the slow and progressive nature of PD, it is essential to replicate the disease course, including behavioral features, to develop precise models (Meredith and Rademacher 2011). However, as *Hspa9*^+/−^ mice showed high mortality 2 days after MPTP injection, we could not examine the associated neuropathological and motor abnormalities for a more extended period. Instead, we observed the early time point after injection, at which time there is relatively little loss of nigrostriatal dopaminergic neurons (Meredith et al. 2008). Notably the accumulation of the active MPTP metabolite MPP^+^ peaks within 90 min and is largely cleared within 12 h (Jackson-Lewis and Przedborski 2007). Therefore, although this acute time point does not fully model the chronic progression of PD, it offers valuable insight into early vulnerability and motor dysfunction. Further study is needed to clarify whether the severe motor abnormalities observed in *Hspa9*^+/−^ mice following MPTP injection are due to enhanced death of dopaminergic neurons or impaired dopaminergic transmission.

In conclusion, our study provides strong evidence that *Hspa9*^+/−^ mice are a suitable model of early-onset PD. Haploinsufficiency of *Hspa9* contributes to the degeneration of dopaminergic neurons by allowing excessive Drp1-dependent mitochondrial fission and ensuing caspase-3 activation, resulting in motor deficits that mimic those observed during the early stages of PD. Further, *Hspa9* insufficiency exacerbates MPTP-induced neurotoxicity. These findings show the involvement of *Hspa9* dysregulation in PD pathogenesis and provide support for further examination of HSPA9 as a potential therapeutic target.

## Supplementary Material

Supplementary Figure_250906.doc

## Data Availability

All of the data generated and analyzed in this study are included in this published article.

## Abbreviations

**Abbreviations:** PD: Parkinson’s disease; AD: Alzheimer’s disease; HSPA9: Heat shock protein family A member 9; TMRE: Tertramethylrhodamine ethyl ester; MPTP: 1-Methyl-4-phenyl-1,2,3,6-tetrahydropyridine; SN: Substantia nigra; STR: Striatum; TH: Tyrosine hydroxylase; Drp1: Dynamin-related protein 1; ROS: Reactive oxygen species; c-caspase-3: Cleaved caspase-3
