# Supplementary material for: Down-regulation of HSPA9 reduces tyrosine hydroxylase-positive neurons in mouse substantia nigra and induces Parkinson’s disease-like motor impairments
Source: Anim Cells Syst (Seoul). 2025 Oct 14;29(1):615–27. doi: 10.1080/19768354.2025.2569875 (PMC12523465; doi:10.1080/19768354.2025.2569875)
Supplement: Supplementary file 1 [file TACS_A_2569875_SM3884.doc]

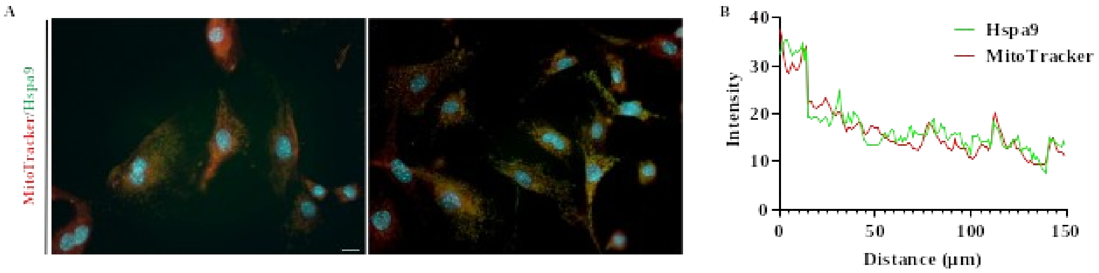

A
MitoTracker/Hspa9
B
Hspa9
MitoTracker
Intensity
Distance (µm)
0
10
20
30
40
0
50
100
150

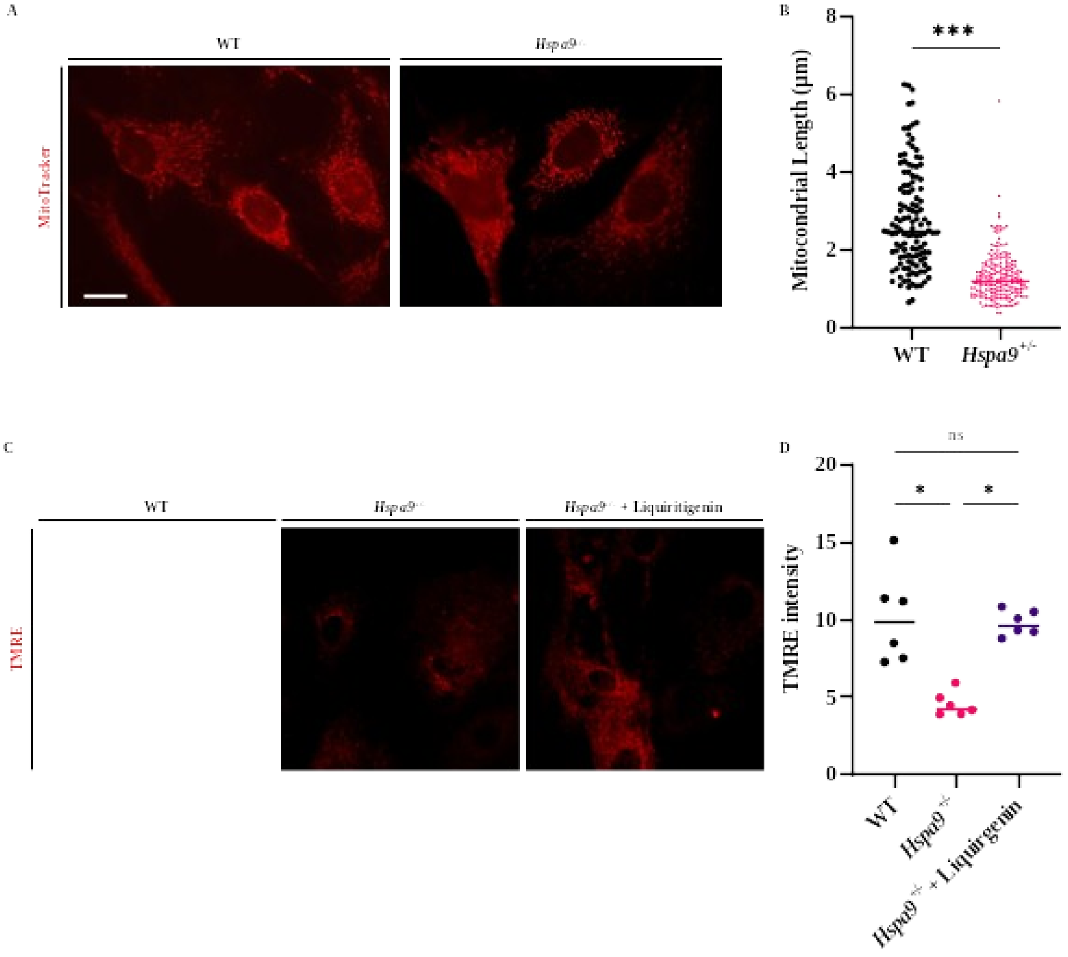
A
WT
Hspa9+/-
MitoTracker
B
Mitocondrial Length (μm)
8
6
4
2
0
***
WT
Hspa9+/-
C
WT
Hspa9+/-
Hspa9+/- + Liquiritigenin
TMRE
D
ns
*
*
TMRE intensity
20
15
10
5
0
WT
Hspa9+/-
Hspa9+/- + Liquirgenin
